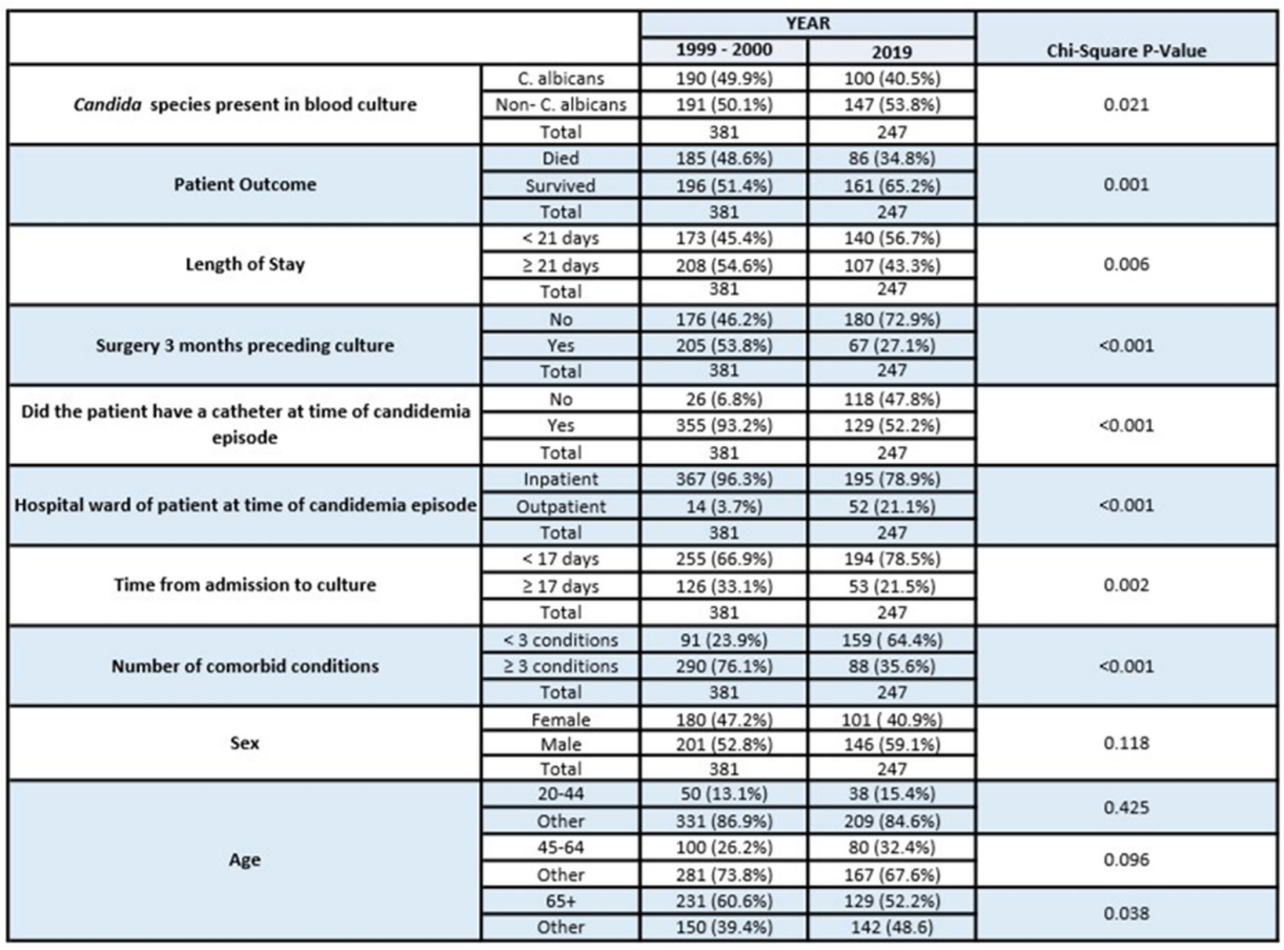# Surveillance of Candidemia in Connecticut: An Epidemiological Comparison Between Two Periods

**DOI:** 10.1017/ash.2021.156

**Published:** 2021-07-29

**Authors:** Johanna Gleason-Vergados, David Banach, Paula Clogher, James Meek

## Abstract

**Background:** Candidemia is the fourth most common bloodstream infection in hospitalized patients in the United States, with an attributable mortality rate between 30% and 50%. Understanding the epidemiology of candidemia is critical due to its prevalence and association with extended hospital stays, high treatment cost, and significant morbidity and mortality. In 2019 the Connecticut Department of Public Health deemed candidemia a mandatory reportable condition and began state-wide surveillance in conjunction with the CDC’s Emerging Infections Program (EIP). Previously, the EIP had conducted population-based statewide surveillance of candidemia in Connecticut from 1998 to 2000, allowing an opportunity to assess how the epidemiology of candidemia has evolved. The goal of this study is to compare state-wide Connecticut EIP candidemia data from 2 periods (1998–2000 and 2019) to identify trends in infections and incidence, providing insight for potential improvements to current prevention measures and treatments. **Methods:** The sample population included all Connecticut residents aged ≥20 who tested positive for a candidemia infection during 1998–2000 and 2019. Patients who had positive blood cultures for *Candida* spp but were < 20 years old or were not Connecticut residents were excluded. Connecticut EIP candidemia case report forms from each time period were compared and matching fields were chosen as variables for univariate analysis to search for statistically significant differences. Selected variables include: Candida species present in blood culture, patient demographics, previous exposures to healthcare settings, length of stay, presence of central venous catheter (CVC), and location of the patient at diagnosis (community vs. hospital onset). De-identified patient-level information was provided by the EIP. **Results:** In total, 381 candidemia episodes from 1998–2000 were compared to 247 episodes in 2019. The proportion of *C. albicans* species in 1998–2000 was 49.9% and declined to 40.5% of cases in 2019 (*P* = .02). Outcomes improved as well, with 65.2% of patients in 2019 having survived compared to 51.4% in 1998–2000 (*P* = .001). Other findings indicate that patients with candidemia in 2019 were less likely to have a central venous catheter, less likely to have undergone a recent surgery, and were more likely to have community-onset infection (all p < 0.05). **Conclusions:** The epidemiology of candidemia has changed over the past 20 years, with significant improvements in patient survival and a shift toward community-onset infections and non-*Albicans* spp. These findings have important implications in designing prevention strategies and optimizing candidemia management, particularly in the community setting where increased intravenous drug use and the availability of home healthcare may be important factors.

**Funding:** No

**Disclosures:** None

**Table 1.**